# Breast Cancer Vaccines: Disappointing or Promising?

**DOI:** 10.3389/fimmu.2022.828386

**Published:** 2022-01-28

**Authors:** Si-Yuan Zhu, Ke-Da Yu

**Affiliations:** ^1^ Department of Breast Surgery, Fudan University Shanghai Cancer Center, Shanghai, China; ^2^ Shanghai Medical College, Fudan University, Shanghai, China

**Keywords:** breast cancer, vaccine, HER2, tumor antigens, E75 peptide vaccine

## Abstract

Breast cancer has become the most commonly diagnosed cancer globally. The relapse and metastasis of breast cancer remain a great challenge despite advances in chemotherapy, endocrine therapy, and HER2 targeted therapy in the past decades. Innovative therapeutic strategies are still critically in need. Cancer vaccine is an attractive option as it aims to induce a durable immunologic response to eradicate tumor cells. Different types of breast cancer vaccines have been evaluated in clinical trials, but none has led to significant benefits. Despite the disappointing results at present, new promise from the latest study indicates the possibility of applying vaccines in combination with anti-HER2 monoclonal antibodies or immune checkpoint blockade. This review summarizes the principles and mechanisms underlying breast cancer vaccines, recapitulates the type and administration routes of vaccine, reviews the current results of relevant clinical trials, and addresses the potential reasons for the setbacks and future directions to explore.

## 1 Introduction

Breast cancer has become the most commonly diagnosed cancer globally, with an estimated burden of 2.3 million new cases in 2020 (1). Breast cancer is heterogeneous and clinically classified into three main subtypes according to the status of estrogen receptor (ER), progesterone receptor (PR), and human epidermal growth factor receptor 2 (HER2): luminal subtype that expresses ER and/or PR, HER2-positive subtype that overexpresses HER2, and triple-negative breast cancer (TNBC) (2). Despite advances in endocrine therapy and anti-HER2 therapy in past decades, relapse and metastasis of breast cancer remain a great challenge in clinical practice. Therefore, innovative therapeutic approaches are still critically in need. In recent years, studies have shown that tumor-infiltrating lymphocyte (TIL) is associated with response to treatment and long-term prognosis in patients with breast cancer (3, 4). Coupled with clinical successes of immune checkpoint blockades (ICB) applied in TNBC and other solid tumors (5–7), intensive interest has arisen in immunotherapy for breast cancer (8, 9).

Immune-based treatment strategies can be divided into passive immunotherapy and active immunotherapy. The anti-HER2 targeted intervention *via* monoclonal antibodies such as trastuzumab and pertuzumab falls under the former category (10, 11). Active immunotherapy mainly refers to cancer vaccines. The cancer vaccine is intended to elicit or boost an anti-tumor immune response by activating autologous immune cells in the patient to induce a therapeutic effect (12, 13). This review summarizes the principles and mechanisms underlying breast cancer vaccines, recapitulates the type and administration routes of vaccine, and reviews the current results of relevant clinical trials. The challenges we face at present and potential directions to explore in the future are discussed in the end.

## 2 Principles of Breast Cancer Vaccine

### 2.1 Immunoediting Throughout Tumor Progression

**Figure 1:**
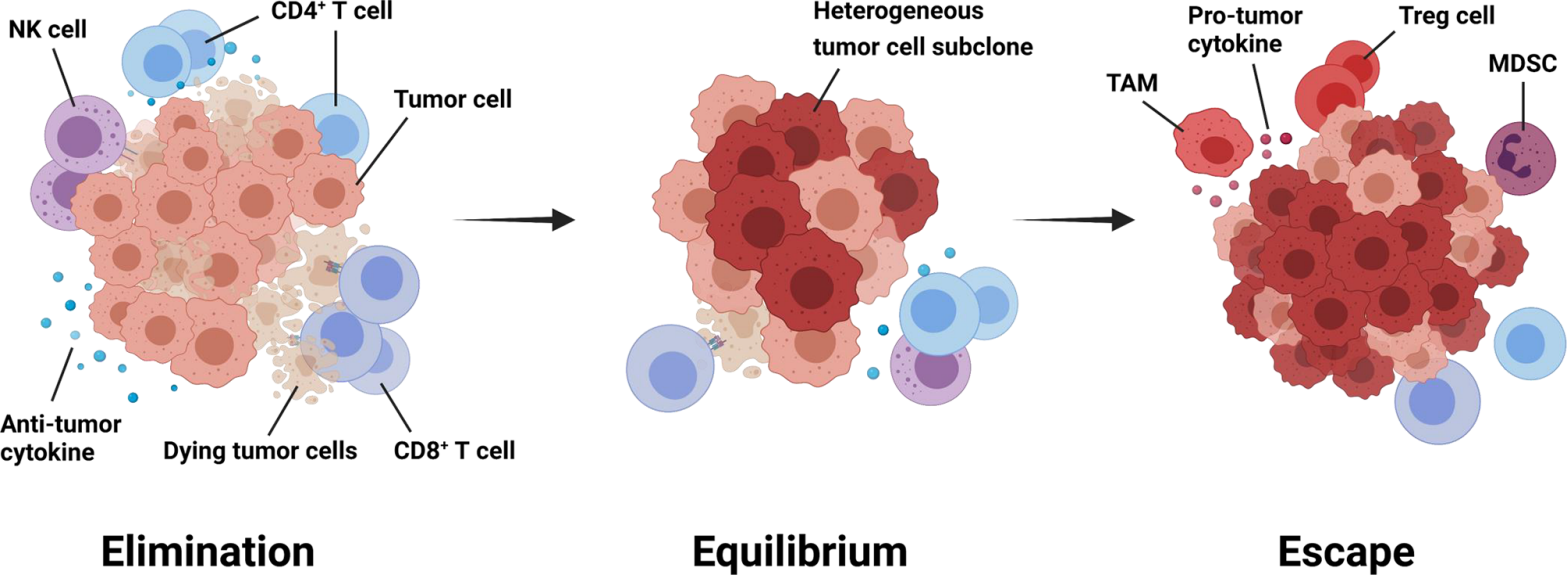
Immunoediting throughout tumorigenesis and progression. Immunoediting generally evolves through three phases: elimination, equilibrium, and escape (14). During the first phase, tumor cells activate anti-tumor immune responses, which mainly performed by CD8^+^ T cells, CD4^+^ T cells, and natural killing cells. The equilibrium phase starts if any tumor subclones survive the selection pressure from the host immunity. Tumor cells can hardly be removed, but meanwhile, their progression is strictly limited in this phase. When shifting to the escape phase, tumor cells with less immunogenicity manage to avoid recognition and attack from anti-tumor immune cells through multiple mechanisms. Besides, an immunosuppressive tumor microenvironment will gradually generate to attenuate anti-tumor immunity and favor tumor progression further. MDSC, myeloid-derived suppressor cell; NK, natural killing; TAM, tumor-associated macrophage; Treg cell, regulatory T cell.

The immune system plays different roles in breast cancer progression during different stage of tumor development. The paradoxical interaction between the tumor and the immune system is referred to as immunoediting, which generally evolves through three phases: elimination, equilibrium, and escape (**Figure 1**) **(** 14). During the elimination phase, incipient tumor cells can activate innate immunity, including maturation of macrophages, natural killing (NK) cells and dendritic cells (DCs). These cells help prime tumor-specific T cells. Thus the adaptive immune response can cooperate with innate immunity to recognize and eradicate these early transformed tumor cells. The equilibrium phase starts if any tumor subclones survives the selection pressure from the host immunity. Tumor cells can hardly be removed, but meanwhile, their progression is strictly limited or even paused because of the delicate balance between tumor growth and the defense effect of the immune system in this phase. However, tumor subclones with less immunogenicity will eventually arise due to tumor cells’ genetic instability and epigenetic modifications (15). These subclones can evade immune recognition and destruction through multiple solutions such as downregulating antigen-presenting molecules and increasing immune checkpoint receptors on the cell surface (16, 17). Therefore, the evolved tumor cells that succeed in escaping constant immunologic pressure will enter the last phase of immunoediting, where the immune system scarcely restrict their progression (18–20).

### 2.2 Immune Cells Recognizing Tumor Antigens

To produce an anti-tumor immune response, the effector immune cells need to recognize tumor antigens presented by tumor cells directly or by antigen-presenting cells (APCs) *via* major histocompatibility complex (MHC) on the cell surface. CD8^+^ and CD4^+^ T cells, which play a core role in the immunoediting process, distinguish these non-self-epitopes of tumor cells displayed by MHC class-I and MHC class-II molecules respectively from normal self-antigens (21–24).

Tumor antigens can be divided into tumor-specific antigens (TSAs) and tumor-associated antigens (TAAs) (25). TSAs are expressed only by tumor cells and not by normal cells. TSAs include oncoviral antigens derived from oncogenic tumor viruses and neoantigens derived from somatic mutations in tumor cells. Therefore there is usually no immune tolerance towards TSAs in humans (26).

TAAs are self-proteins commonly expressed in both tumors and normal tissues, while their expression patterns in tumor cells are abnormal (27). This category includes overexpressed antigens such as HER2 and mucin-1 (MUC-1), tissue differentiation antigens such as carcino-embryonic antigen (CEA), and tumor germline antigens like melanoma-associated antigen (28). The majority of tumor antigens that have been studied in breast cancer vaccines so far are the HER2 protein and other HER2-derived peptides (29, 30). In humans, the HER2 protein is generally expressed during fetal development and is weakly detectable in the epithelial cells of many normal tissues in adults (31). Thus immune tolerance to HER2 has usually been established already. In fact, despite the existence of immune tolerance, humoral and cellular immunity against HER2 have been detected in some of breast cancer patients due to the high immunogenicity of the antigen (32, 33). However, the level of the pre-existed anti-HER2 immunity is usually too low to induce an evident therapeutic effect. Therefore, vaccines targeting HER2-related antigens need to overcome the established tolerance to boost an immune response that is strong and durable enough (31). Various strategies, including using novel immunoadjuvants, applying dominant or subdominant epitopes, and altering the natural structure of peptides, have been investigated in breast cancer vaccines to circumvent immune tolerance.

### 2.3 Tumor Cells Attenuating Anti-Tumor Immunity

To successfully escape immunosurveillance, tumors manage to suppress the host immunity both systemically and locally (34). As mentioned above, when the elimination phase gradually shifts to the escape phase, the immunosuppressive effect will outweigh the antitumoral response in the relatively advanced stage of the disease. During this shift, suppressive immune cells, including regulatory T (Treg) cells, tumor-associated macrophages (TAMs), and myeloid-derived suppressor cells (MDSCs), become increasingly prevalent in the tumor microenvironment (TME) and the draining lymph nodes of the tumor and even appear in peripheral blood (35–39). Increased number of these immunosuppressive cells generally correlates to inferior prognosis (38–42). Moreover, the number and the activity of the cytotoxic lymphocytes (CTLs) and NK cells in the TME are reduced so that the antitumoral response will be further undermined (43–46).

In addition to the transformation of immune cell composition in the TME, cytokines are also involved in generating an immunosuppressive microenvironment in favor of tumor progression (47). For instance, upregulation of the DC-derived cytokine TGFβ promotes the proliferation of Treg cells (48), and Treg cells will correspondingly downregulate the co-stimulatory molecules such as CD80 and CD86 on DCs required for CTL priming (45). The cytokine interleukin-2 (IL-2), which is necessary for CTL activation, can bind to Treg cells at a higher affinity, leaving the CTLs in starvation (46). Moreover, adenosine produced by Treg cells has an immune inhibitory effect on the effector T cells (49, 50). The inhibitory cytokine IL-10 and TGFβ secreted by TAMs are also capable of blocking the function of CTLs (51, 52) and suppress the production of anti-tumor cytokine IL-12 (53).

Furthermore, immune checkpoint receptors such as programmed cell death receptor 1 (PD-1) and cytotoxic T lymphocyte antigen 4 (CTLA-4) are found to be upregulated in tumor progression. PD-1 is the counter-receptor of programmed cell death ligand 1 (PD-L1) (54). In patients with different malignant tumors, high levels of PD-1 expression are detected in TILs, including tumor-specific T cells, and PD-L1 is upregulated in tumor cells and APCs simultaneously. Engagement of PD-L1 and PD-1 results in T cell dysfunction and apoptosis so that the tumor cells can avoid destruction from T cells (55, 56). CTLA-4 is found in the intracellular compartment in resting T cells and it will be transported to the cell surface once the T cell is stimulated (57). It can block the co-stimulatory signals, which is essential for T cell activation, through binding the transmitting molecules CD80 and CD86 on DCs and B cells to prevent the immune response from over-amplification (58). ICB blocks the inhibitory receptors such as PD-1/PD-L1 and CTLA-4, allowing effector T cells to attack the tumor (59). The efficacy of ICB for breast cancer has recently been evaluated. Monoclonal antibody atezolizumab targeting PD-L1 successfully prolonged progression-free survival (PFS) among patients with metastatic TNBC in the IMpassion130 trial (7). However, the same drug failed to show a significant improvement in PFS for advanced HER2-positive breast cancer in combination with trastuzumab emtansine in the KATE2 trial (60).

Collectively, the suppressive immune cells, the cytokines, the metabolites, and immune checkpoint molecules together constitute a complex network of immune suppression that facilitates immune escape and attenuates anti-tumor immunity.

## 3 Approaches of Breast Cancer Vaccine

Strategies of vaccination involve optimization of vaccine regimens and administration routes. Breast cancer vaccines can be divided into different types based on platforms and formulations. Nevertheless, they all need to make the targeted antigen recognized by the autologous immune system to induce a therapeutic effect. Adjuvant of the vaccine plays a vital role as they are able to enhance antigen immunogenicity and regulate the immune response. Additionally, administration routes have different influences on the delivery of targeted antigens to DCs. We will briefly review the types of breast cancer vaccines and introduce the adjuvants and administration routes applied currently.

### 3.1 Types of Breast Cancer Vaccine

**Figure 2:**
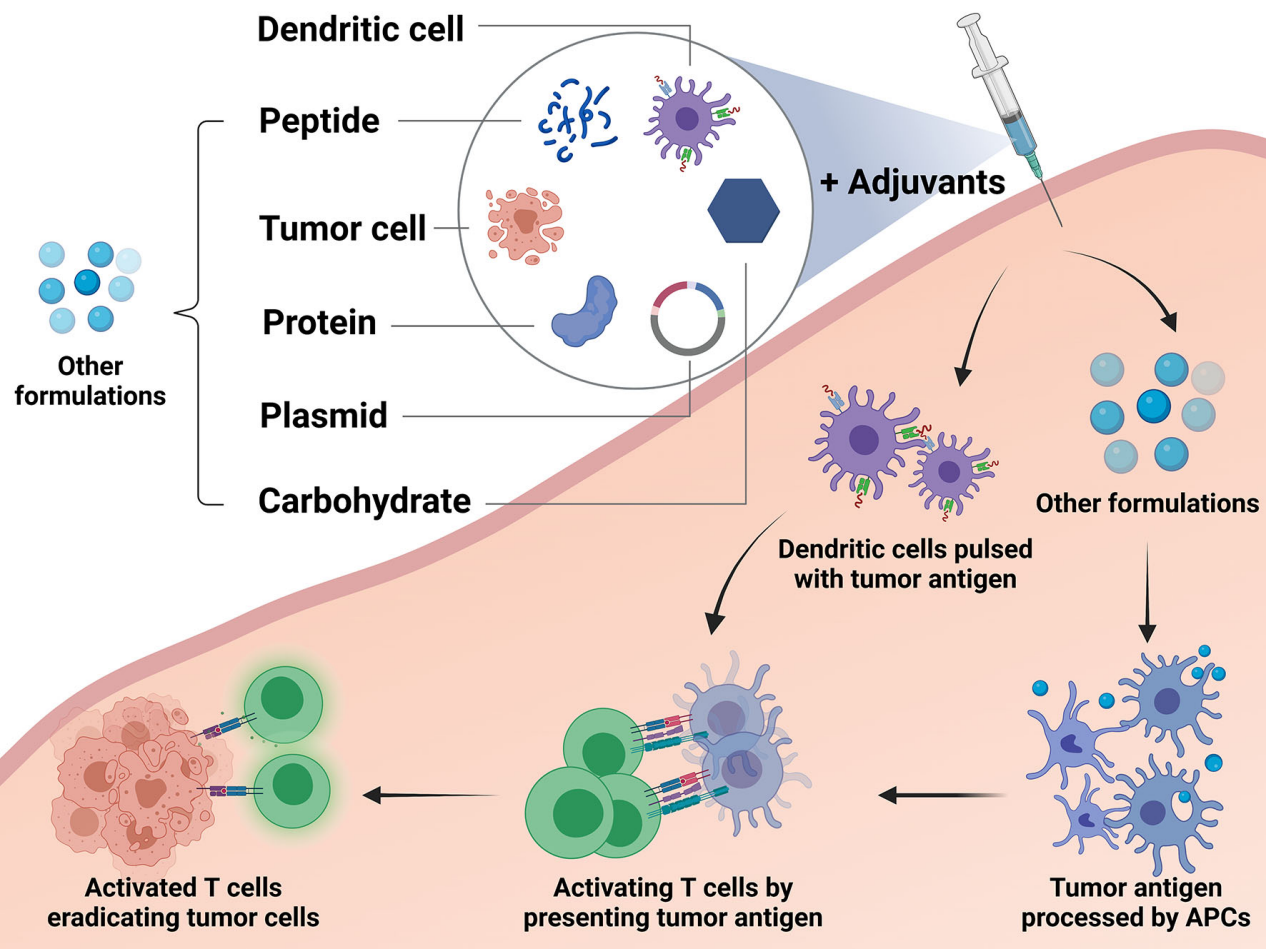
Different types of breast cancer vaccines and their mechanisms. The studied breast cancer vaccines can be divided into the following types according to their formulations and approaches: peptide vaccine, protein-based vaccine, carbohydrate antigen vaccine, DNA-based vaccine, dendritic cell-based (DC-based) vaccine, and tumor cell vaccine. DC-based vaccines utilize *ex vivo* generation of DCs loaded with tumor antigens or transfected to express tumor antigens. These cells process the antigens and present them to T cells directly by themselves in order to activate an immune response. Except the dendritic cells, other formulations applied in the vaccines, including peptide, protein, plasmid, carbohydrate and tumor cell, need to stimulate the autologous antigen presenting cells (APCs). Then the autologous APCs will activate the effector immune cells to boost an anti-tumor reaction.

Currently, the most common vaccination approach for breast cancer is to utilize peptides derived from tumor antigens. Vaccination of tumor antigen-related protein and carbohydrate has also been explored for long. Tumor cell-based vaccine is one of the traditional methods, while DNA-based and DC-based vaccines represent novel modalities in this field. A different formulation of vaccines and their mechanisms of action are depicted in **Figure 2**.

#### 3.1.1 Peptide Vaccine

Delivering MHC class-I restricted peptide epitopes to activate immune responses against the specific tumor antigen is one of the most common strategies applied for breast cancer. The peptide injected will be processed and presented by APCs to prime immune effector cells, which will then seek out and eradicate cancer cells expressing the shared antigen (61). Compared to other formulations, short amino acid peptides are simple and cheap to manufacture and relatively stable when transported, which makes large-scale manufacture and transportation possible (62). However, the individual peptide is usually limited to certain human leukocyte antigen (HLA) subtypes and thus patients who do not express the common HLA types cannot be treated with the vaccine (63). In addition, the usual MHC class-I binding peptides do not have a strong ability to activate CD4^+^ helper T cells, which may cause limited activation of CD8^+^ cytotoxic T cells and transience of immune responses (64). This issue might be partly overcome by using synthetic peptides that are long enough to include multiple MHC class-I and class-II epitopes. Such peptides containing 23-45 amino acids might lead to superior T cell stimulation through a more efficient processing and presentation pathway (65).

#### 3.2.2 Protein-Based Vaccine

The protein-based vaccine is developed with the whole or shortened fragment of tumor antigen protein whose amino acid sequence is much longer than peptides (64). It enables uptake, processing, and presentation of multiple MHC class-I and class-II peptide epitopes and is not HLA restricted. But the presentation process might be less efficient, and the response to this kind of vaccine is hard to measure due to lack of a specific marker (66).

#### 3.1.3 Carbohydrate Antigen Vaccine

Carbohydrate antigens abnormally expressed by tumor cells can also be distinguished by immune cells. Hence, such carbohydrate antigen becomes an ideal candidate to incorporate in a cancer vaccine. For instance, Sialy-Tn (STn), a disaccharide carbohydrate associated with MUC-1, is expressed uniquely on the cell surface of a variety of cancer cells, including breast cancer (67). Immunization with STn demonstrated tumor regression and prolonged survival in animal studies, and the cancer vaccine towards STn was correspondingly developed (68).

#### 3.1.4 Tumor Cell Vaccine

It is one of the earliest approaches of the cancer vaccine to use whole tumor cells or products of tumor cell lysis to stimulate an immune response (64). It is based on a pool of unknown antigens derived from autologous or allogeneic tumor cells, and thus a polyvalent immune response will be triggered. The tumor cells are modified to secret cytokines or express co-stimulatory molecules in order to enhance the antigen-presenting ability in some vaccines (69, 70). The disadvantage of the tumor cell vaccine lies in that these vaccines contain endogenous cellular antigens and may cause an autoimmune reaction. There is also a lack of a standardized method for preparing tumor cell vaccines (63).

#### 3.1.5 DNA-Based Vaccine

The DNA-based breast cancer vaccine uses the DNA sequence encoding tumor antigens, which are usually delivered in the forms of plasmids or vectors. The DNA sequence will be incorporated by APCs and translated into the tumor antigen, which will then be processed for presentation for immune cells to stimulate an antigen-specific immunity (71). DNA-based vaccines are easy to construct in large quantities and store at a low cost. However, the immunogenicity is not strong enough due to low efficiency of plasmids uptake and antigen expression (63, 71).

#### 3.1.6 DC-Based Vaccine

DCs are a heterogeneous population of APCs that efficiently take up antigens and then process and present the antigens to CD4^+^ and CD8^+^ T cells after migrating to lymph nodes. NK cells and B cells can also be stimulated by DCs (61). The DC-based vaccines usually utilize *ex vivo* generation of DCs loaded with tumor antigens or transfected to express tumor antigens. Monocytes and CD34+ progenitor cells have been tested, and antigens including complex tumor lysates and multiple MHC class-I and class-II peptides have been explored in studies (62). Some vaccines require inoculation in lymph nodes and the DCs delivered can activate the immune cells directly. The production of DC-based vaccines can be technically demanding due to the individualized *ex vivo* process for the maturation of DCs (64). It is therefore difficult to compare trials with a single clinical trial arm and individualized vaccination patterns.

#### 3.1.7 DC-Tumor Cell Fusion Vaccine

One of the efforts to improve the DC-based vaccination strategy is the fusion of DCs with tumor cells. DC-tumor cell hybrids can be created by exposing DCs and tumor cells in polythelene glycol (72). Tumor cells can also be transfected with a viral fusogenic membrane glycoprotein and pelleted with DCs to achieve a DC-tumor hybrid (73). Besides, electrofusion technique has been applied in this strategy (74). Compared with DCs pulsed with single antigens, DC-tumor cell fusion is able to present the entire repertoire of tumor antigens from the parental tumor cell to activate both the MHC class-I and class-II pathways (75). Nevertheless, this kind of vaccine is even harder to produce compared to the DC-based vaccine pulsed with peptides.

### 3.2 Adjuvants for Breast Cancer Vaccine

Adjuvants are substances that enhance antigen immunogenicity and elicit an immune response when inoculated with antigens (76). Mechanisms of most adjuvants include slowing release of antigens, promoting antigen uptake and presentation of APCs and stimulating proliferation of DCs and macrophages (77–79). In prophylactic vaccines designed for infectious diseases, classical adjuvants, such as alum, mainly induce the type 2 T helper cell-dependent humoral immunity instead of type 1 T helper cell responses that directly destruct tumor cells (80). Different types of adjuvants used in cancer vaccines are listed in **Table 1**.

**Table 1:** Major types of adjuvants for breast cancer vaccine and their functions.

Types of Adjuvants	Examples	Functions
Cytokines	GM-CSF, IL-12	Promoting the maturation and activation of DCs and enhancing antigen uptake and presentation
Microbes and microbial derivatives	BCG, CpG, MPL, poly I:C	Activating DCs through toll-like receptor ligands
Mineral salts	Alum	Enhancing antibody production by plasma cells
Oil emulsions or surfactants	AS02, Montanide, QS21	Decelerating release of antigens and stimulating local DCs at the injection site
Particulates	AS04, polylactide co-glycolide	Functioning as an antigen carrier and enhancing antigen uptake and presentation
Viral vectors	Adenovirus, fowlpox	Delivering antigens and activating DCs through toll-like receptor ligands

AS, adjuvant system; BCG, Bacillus Calmette-Guérin; CpG, cytosine-phosphate diester-guanine; DC, dendritic cell; GM-CSF, Granulocyte-macrophage colony-stimulating factor; IL, interleukin; MPL, monophosphoryl lipid A; QS21, a plant extract derived from Quillaja saponaria.

Granulocyte-macrophage colony-stimulating factor (GM-CSF) is a secreted cytokine that has been widely used as an adjuvant in breast cancer vaccines. It has been shown to be capable of triggering the maturation of myeloid cells such as granulocytes and macrophages and promoting the expansion and activation of DCs (81, 82). Several breast cancer vaccines containing GM-CSF induced detectable immune responses in clinical trials (83–87). And in melanoma patients, locally addition of GM-CSF modestly increased the immune response towards the vaccinated antigen (78, 88). However, in other studies, it was also observed that GM-CSF might be associated with a lower degree of T cell responses and induction of inhibitory MDSCs (89, 90). Therefore, the application of GM-CSF as an adjuvant in cancer vaccines still needs further investigation.

Another popular strategy for adjuvants adopted in DNA-based cancer vaccines is utilizing recombinant viral vectors. Recombinant viral vectors, which usually function as a delivery vehicle for the antigen, can boost immune response as well in that they always contain more or less toll-like receptor (TLR) ligands and pattern recognition receptor ligands to activate DCs (91). The TLR agonists are also able to enhance CD8^+^ T cell activation and prevent T cell from exhausting (92, 93). The main drawback of such an adjuvant is that the vectors also have other sequences capable of competing with the inserted sequence of targeted antigens (94).

Nevertheless, difficulty exists when comparing different adjuvant strategies for cancer vaccines since the effects of adjuvants might vary with vaccine formulations, targeted tumor antigens, immunization schedule, and route of administration. Therefore relevant studies on the optimization of adjuvants for breast cancer vaccines are urgently necessary at present.

### 3.3 Administration Routes of Breast Cancer Vaccine

**Figure 3:**
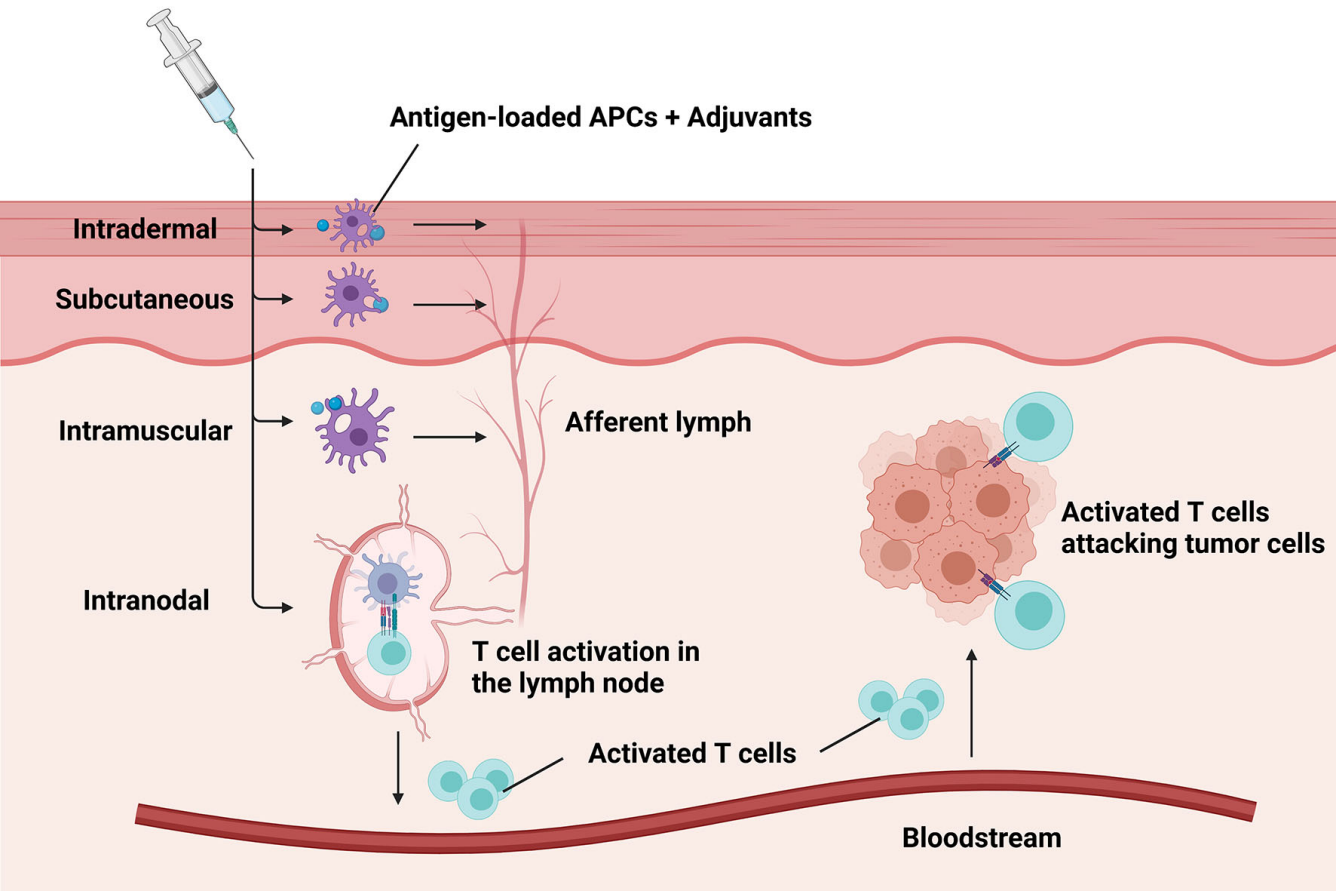
Different administration routes of breast cancer vaccines. Major administration routes of breast cancer vaccines include intradermal, subcutaneous, intramuscular, and intranodal injection. The preferred routes depending on the type of the delivered antigens help effectively present the antigens to autologous antigen-presenting cells (APCs). Then the antigen-loaded APCs transfer to lymph nodes to prime T cells through afferent lymph. Subsequently, activated T cells transport into tumorous tissue with the aid of the bloodstream to eradicate tumor cells.

Administration routes of cancer vaccines help effectively present the antigens to autologous APCs. Different preferred routes were applied for cancer vaccines of different types (**Figure 3**). Several peptide vaccines targeting HER2 have adopted intradermal vaccination strategies as there is a dense network of cutaneous DCs (83–85). Studies demonstrated that intradermal inoculation with low doses of the peptide was safe and stimulated antigen-specific T cell responses in the majority of the healthy population (95). The subcutaneous injection was also practiced in a variety of different breast cancer vaccines and achieved immune responses. However, large volumes of antigen delivered subcutaneously with adjuvants might cause severe injection-site reactions with occasional sterile abscess formation (96), which may lead to discontinuation of vaccination procedure or reduction of vaccine doses. In addition, intramuscular administration was often used to deliver vectors or plasmids for some DNA-based vaccines (97–99). By contrast, some DC-based vaccines required intranodal injection in order to prime the T cells existing in the lymph node directly.

An important factor to consider is how administration routes of vaccines affect the circulating and homing process of T cells towards the cancer-infiltrated tissues. Recent studies showed that intranasal immunization with DCs from the lung parenchyma was able to trigger homing properties on induced CD8^+^ T cells to the mucosa (100). Much more work is necessary to establish valid rules regarding the delivery routes of cancer vaccines.

## 4 Clinical Trials of Breast Cancer Vaccine

Some breast cancer vaccines managed to elicit detectable immune responses and demonstrate good tolerance in early trials. Nevertheless, none of them has demonstrated significant clinical benefits in the following phase 3 trials. The Theratope^®^ (STn) vaccine applied in the metastatic setting and the NeuVax™ [Nelipepimut-S (NPS), or E75] vaccine applied in the adjuvant setting both failed to bring clinical benefits in their phase 3 study despite their early success (101, 102). We summarize the current results of clinical trials evaluating breast cancer vaccines according to the antigen they target in the following paragraphs. Major clinical trials targeting HER2-related antigens and non-HER2-related antigens are listed in **Tables 2** and **3**, respectively.

**Table 2:** Major clinical trials on breast cancer vaccines targeting HER2-related antigens.

Clinical Trial Reference	Trial Phase	Setting	Targeted Tumor Antigen	Design and Arms	Breast Cancer Subtype	Primary Objectives	Outcomes
PRESENT Trial	III	Adjuvant	HER2-derived peptide E75	Vaccination Arm: E75 + GM-CSF (N=376)	HLA-A2/A3+, HER2 low-expressing (IHC 1/2+), node-positive	DFS	RR at 16.8 months interim analysis: 9.8% (vaccinated group) versus 6.3% (control group) (P = 0.07). Based on these data, the study was terminated for futility.
NCT01479244				Control Arm: Placebo + GM-CSF (N=382)			
**(** 102 **)**							
US Military Cancer Institute Clinical Trials Group Study I-01 and I-02 **(** 103 **)**	I/II	Adjuvant	HER2-derived peptide E75	Vaccination Arm: E75 + GM-CSF of different doses (N=108)	HLA-A2/A3+, HER2-expressing, node-positive or high-risk node-negative	Safety, optimal dosing of immune response	Five-year DFS: 89.7% (vaccinated group) versus 80.2% (control group) (P = 0.08). Toxicities were minimal.
				Control Arm: Observation (N=79)			
NCT01570036 **(** 104 **)**	II	Adjuvant	HER2-derived peptide E75	Vaccination Arm: E75 + GM-CSF + trastuzumab (N=136)	HLA-A2/A3+, HER2 low-expressing (IHC 1/2+), node-positive	DFS	The estimated 24-month DFS: 89.8% (vaccinated group) versus 83.8% (control group) (P= 0.18).
				Control Arm: Placebo + GM-CSF + trastuzumab (N=139)			
NCT00524277 **(** 105, 106 **)**	II	Adjuvant	HER2-derived peptide GP2	Vaccination Arm: GP2 + GM-CSF (N=89)	HLA-A2+, HER2-expressing, node-positive or high-risk node-negative	DFS, RR	The estimated 5-year DFS: 88% (vaccinated group) versus 81% (control group) (P = 0.43); 100% (HER2 3+ vaccinated patients) versus 89% (HER2 3+ placebo patients) (P=0.03).
				Control Arm: GM-CSF alone (N=91)			
US Military Cancer Institute Clinical Trials Group Study I-04 **(** 84 **)**	I	Adjuvant	HER2-derived peptide GP2	Single arm: GP2 + GM-CSF of different doses (N=18)	HLA-A2+, HER2-expressing, node-negative	Safety, immune response	Immune response was induced in all the enrolled patients. Toxicities were minimal.
NCT00524277 **(** 107 **)**	II	Adjuvant	HER2-derived peptide AE37	Vaccination Arm: AE37 + GM-CSF (N=153)	HLA-A2+, HER2-expressing, node-positive or high-risk node-negative	RR	RR at 25-month median follow-up: 12.4% (vaccinated group) versus 13.8% (control group) (P=0.70).
				Control Arm: GM-CSF alone (N=145)			
US Military Cancer Institute Clinical Trials Group Study I-03 **(** 85 **)**	I	Adjuvant	HER2-derived peptide AE37	Single arm: AE37 + GM-CSF of different doses (N=15)	HLA-A2+, HER2-expressing, node-negative	Safety, immune response	Immune response was induced in all the enrolled patients. Toxicities were minimal.
NCT00399529 **(** 108 **)**	II	Metastatic	HER2	Single arm: HER2 GM-CSF-secreting tumor cell vaccine + cyclophosphamide + trastuzumab (N=20)	Stage IV, HER2-expressing	Safety, CBR	CBR at 6 months and 1 year was 55% and 40%, respectively. Toxicities were minimal.
NCT00140738 **(** 109 **)**	I/II	Metastatic	HER2	Single arm: recombinant HER2 protein + AS15 (N=40)	Stage IV, HER2-expressing	Safety, CBR	Clinical activity was observed with 2/40 objective responses and prolonged stable disease for 10/40 patients. Immunization was associated with minimal toxicity.
NCT02061332 **(** 110 **)**	II	Neoadjuvant	HER2	Single arm: HER2 dendritic cell vaccine with different routes (N=27)	HER2-expressing DCIS or early invasive breast cancer	Safety, immune and clinical response	Vaccination by all injection routes was well tolerated. There was no significant difference in immune response rates by vaccination route.

CBR, clinical benefit rate; DCIS, ductal carcinoma in situ; DFS, disease-free survival; GM-CSF, granulocyte-macrophage colony-stimulating factor; HER2, human epidermal growth factor receptor 2; HLA, human leukocyte antigen; IHC, immunohistochemistry; RR, recurrence rate.

**Table 3:** Major clinical trials on breast cancer vaccines targeting non-HER2-related antigens.

Clinical Trial Reference	Trial Phase	Setting	Targeted Tumor Antigen	Breast Cancer Subtype	Primary Objectives	Outcomes
NCT00003638 **(** 101 **)**	III	Metastatic	STn	Stage IV	TTP, OS	TTP: 3.4 months (treatment group) versus 3.0 months (control group) (P=0.35). Median OS: 23.1 months (treatment group) versus 22.3 months (control group) (P=0.91).
Miles DW, et al. **(** 111 **)**	II	Metastatic	STn	Stage IV	Safety, immune and clinical response	Clinical activity was observed with 2/18 minor responses and stable disease for 5/18 patients. Toxicities were minimal.
NCT00179309 **(** 112 **)**	II	Metastatic	Mucin-1, CEA	Stage IV	PFS	Median PFS: 7.9 months (vaccinated arm) versus 3.9 months (control arm) (P=0.09).
Svane IM, et al. **(** 113 **)**	II	Metastatic	p53	Stage IV HLA-A2+	Safety, immune and clinical response	Clinical activity was observed with 8/19 stable disease or minor regression with 11/19 progressive disease. Toxicities were minimal.
Domchek SM, et al. **(** 114 **)**	I	Metastatic	hTERT	Stage IV HLA-A2+	Immune response	High immune response was observed in 9/16 patients and non/low response was seen in 7/16 patients.
NCT00807781 **(** 99 **)**	I	Metastatic	Mammaglobin-A	Stage IV HLA-A2/A3+	Safety, immune response	No serious adverse events and a significant increase in the frequency of MAM-A specific CD8^+^ T cells after vaccination (0.9% vs. 3.8%, P<0.001) was observed.
Avigan D, et al. **(** 115 **)**	I	Metastatic	Multiple antigens	Stage IV	Safety, clinical response	No significant toxicity or autoimmunity. Clinical activity was observed with 2/10 disease regression and 1/10 disease stabilization.

CEA, carcino-embryonic antigen; HLA, human leukocyte antigen; hTERT, human telomerase reverse transcriptase; OS, overall survival; PFS, progression-free survival; STn, Sialyl-Tn; TTP, time to progression.

### 4.1 Vaccines Targeting HER2-Related Antigens

Breast cancer vaccines deliver HER2 or HER2-related antigens through different approaches and formulations. In this field, several peptide vaccines have been studied extensively in phase 2-3 clinical trials. We will introduce the vaccines targeting HER2-related antigens in the order of their types.

#### 4.1.1 Peptide Vaccine—E75

E75 (Nelipepimut-S) vaccine is one of the most extensively studied breast cancer vaccines against HER2. It consists of HLA-A2/A3-restricted, MHC class-I, extracellular HER2-derived peptide E75 and the immunologic adjuvant GM-CSF. In a phase 1 trial initiated in the adjuvant setting, the E75 vaccine was administered to the disease-free patient with any level of HER2 expression [immunohistochemistry (IHC) 1-3+]. An immune response with good tolerance was demonstrated (83). The monthly intradermal dose of 1000mg E75 and 250mg of GM-CSF for 6 months was determined to be optimal (116). In the following phase 2 adjuvant study, 195 patients were randomly assigned to the vaccination arm or the control arm. At the conclusion of 5-year follow up, the disease-free survival (DFS) rate was 89.7% for vaccinated patients and 80.2% for control patients (P=0.08) (103, 116). Interestingly, vaccinated patients with relatively low expression of HER2 (IHC 1-2+) demonstrated a more robust immune response compared to those with higher levels of HER2 expression (IHC 3+), suggesting the possibility of immunologic tolerance to HER2 in some patients with tumors expressing high levels of HER2 (117).

Based on these promising data, the multicenter double-blinded phase 3 PRESENT trial was undertaken in patients with node-positive breast cancer with IHC 1-2+ HER2 expression in the adjuvant setting (102). In total, 758 disease-free patients were randomized to receive NeuVax™ or placebo. The primary endpoint was 3-year DFS. However, this trial was terminated due to futility when an interim analysis, which was triggered after 70 qualifying DFS events occurred, failed to show a significant difference in DFS with vaccination. There were even more DFS events in the vaccinated group than in the control group. Still, the deaths, second cancers, and clinical recurrences were similar at 16.8 months median follow-up.

When combined with anti-HER2 targeted therapy, the efficacy of E75 vaccine in patients with low expression of HER2 (IHC 1-2+) was evaluated in a recently conducted phase 2 adjuvant trial (104). A total of 275 patients were randomized to receive E75 or placebo after receiving 1-year standard trastuzumab-based anti-HER2 treatment. At a median follow up of 25.7 months, estimated DFS did not significantly differ between the vaccination arm and the control arm (P = 0.18). But significant improved DFS was seen in patients with TNBC (IHC 1-2+ and hormone receptor-negative) in a planned exploratory analysis (P = 0.01). This study reflects that the HER2-derived peptide vaccines might be effective when used in parallel to or combined with trastuzumab-based anti-HER2 targeted therapy.

As for HER2 overexpression (IHC 3+) patients, the efficacy of E75 remains ambiguous in that the majority of the HER2 overexpression patients enrolled in the existing trials did not receive trastuzumab as standard anti-HER2 therapy.

#### 4.1.2 Peptide Vaccine—GP2

Although the results of NeuVax™ are not satisfying, new promise comes from other latest studies. GP2 is another HLA-A2/A3-restricted, MHC class-I, an immunogenic peptide derived from the transmembrane domain of HER2. While GP2 has a lower affinity to HLA-A2 than E75, it is as efficacious in inducing a CD8^+^ T cell response (118). The GP2 vaccine demonstrated a good safety profile and managed to generate GP2-specific T cell responses and GP2-specific delayed-type hypersensitivity (DTH) responses when administered with GM-CSF in a phase 1 adjuvant trial (84). In the following phase 2 adjuvant trial that enrolled 180 patients with tumors expressing HER2 (IHC 1-3+), no significant benefit in DFS in the vaccination group compared with the control group (88% vs. 81%, P=0.43) after a 34-month median follow-up was observed (105). A subgroup analysis showed that HER2-positive (IHC 3+) patients had no recurrences with a trend towards improved DFS in the vaccinated group as compared to the control group (100% vs. 87.2%, P=0.052) (119). Encouraging results came from the final analysis of this trial, which demonstrated that the GP2 vaccine reduced the recurrence rate to 0% in HER 3+ patients, who have received a standard course of trastuzumab after surgery. The estimated 5-year DFS rate in the 46 HER2 3+ vaccinated patients, if the patient completed the primary immunization series, was 100% versus 89.4% in the 50 placebo patients (p=0.034) (106).

#### 4.1.3 Peptide Vaccine—AE37

In addition to E75 and GP2, AE37 is another HER2-related peptide vaccine used in the adjuvant setting of breast cancer. It is an Ii-Key hybrid of AE36, which is derived from the intracellular domain of HER2. The modification was conducted to improve the binding potency of the epitope (120). Unlike E75 and GP2, AE37 is an MHC class-II epitope that mainly induces CD4^+^ T cell activation. Low toxicity and favorable immune response were demonstrated in a phase 1 trial (85). Levels of Treg cells were measured and found to decrease after vaccination as AE37 stimulates CD4^+^ helper T cell response (121). In a phase 2 trial of clinically disease-free patients expressing any degree of HER2 (IHC 1-3+), AE37 plus GM-CSF and GM-CSF alone were randomly administered to 153 and 145 patients, respectively (107). The DFS rate was 87.6% in the vaccine group and 86.2% in the control group (P=0.70) after a median follow-up of 30 months. In planned subset analyses of patients with IHC 1-2+ HER2-expressing tumors, DFS was 86.8% in vaccinated patients and 82.0% in control patients(P=0.21). Interestingly, TNBC patients (IHC 1-2+ and hormone receptor-negative) demonstrated a DFS rate of 84.0% in the vaccine group and 64.0% in the control group (P=0.12), suggesting AE37 vaccination may lead to clinical benefits in patients with low HER2-expressing tumors, specifically TNBC.

#### 4.1.4 Protein-Based Vaccine

As for the protein-based vaccine, in a phase 1 study, 29 patients with stage II-IV HER2-overexpressing breast and ovarian cancer were vaccinated with the intracellular domain of HER2 (amino acids 676-1255) plus GM-CSF (86). As a result, 89% of the patients developed HER2-specific T cell immunity, and HER2-specific antibody immunity was observed in 82% of the patients. Cellular immunity was maintained for 9-12 months after completion of immunization in over half of the patients.

In another phase 1 trial, another recombinant HER2 protein with adjuvant AS15 was administered to 61 trastuzumab-naive patients with stage II-III HER2-overexpressing breast cancer after surgical resection and adjuvant therapy (122). Association was found between the vaccination dose, the immunization schedule, and the prevalence of HER2-specific humoral responses. The HER2-specific immunity was maintained for over 5 years in 6/8 patients who received the highest dose of vaccination. In the metastatic setting, the same vaccine regimen was administered to 40 HER2-overexpressing metastatic breast cancer patients as first or second-line therapy following response to trastuzumab-based treatment as maintenance (109). The vaccine was well-tolerated and clinical activity was observed with 2 objective responses and prolonged stable disease for 10 patients.

#### 4.1.5 Tumor Cell Vaccine

A HER2-positive tumor cell vaccine that was modified to secret GM-CSF has been evaluated in clinical trials. A total of 28 patients with metastatic breast cancer received the vaccine in combination with cyclophosphamide and doxorubicin to test the hypothesis that the two chemotherapy drugs can enhance vaccine-induced immunity (87). HER2-specific DTH and antibody responses were observed with low toxicity in most patients, and the optimal dose of chemotherapy was cyclophosphamide at 200mg/m^2^ and doxorubicin at 350mg/m^2^. The vaccine was administered to 20 HER2-positive metastatic breast cancer patients with a low dose of cyclophosphamide (300mg/m^2^) and weekly trastuzumab in another single-arm clinical trial (108). Augmented HER2-specific immunity was also detected by enhanced DTH and CD8^+^ T cell responses.

#### 4.1.6 DNA-Based Vaccine

In a pilot phase 1 study, the DNA vaccine encoding a full-length signaling-deficient version of HER2 was injected together with GM-CSF and IL-2 to 8 patients with metastatic HER2-positive breast cancer who were also treated by trastuzumab (97). Treatment for 2 patients was discontinued after one vaccine cycle due to rapid tumor progression or disease-related complications. The vaccine was proven to be safe in the trial. Although no T cell responses towards HER2 were observed immediately after vaccination, a significant increase of MHC class-II restricted T cell responses to HER2 was detected at long-term follow-up.

Another multicenter phase 1 study using a DNA vaccine named V930 involved 33 patients with stage II-IV solid tumors expressing HER2 or CEA (98). V930 contained equal amounts of plasmids expressing the extracellular and transmembrane domains of HER2 and a plasmid expressing CEA fused to the B subunit of Escherichia coli heat-labile toxin. Patients were randomly assigned to receive V930 alone or V930 followed by V932, another adenovirus subtype-6 viral vector vaccine coding for the same antigens. In spite of good tolerance in both approaches, no measurable cell-mediated immune response to CEA or HER2 was either detected.

Currently, ongoing clinical trials (NCT00393783, NCT00436254) are evaluating the safety and immunologic activity of DNA-based vaccines encoding different versions of HER2-derived protein in treating HER2-overexpressing breast cancer.

#### 4.1.7 DC-Based Vaccine

The efficacy of a DC-based vaccine towards HER2 was examined in patients with HER2-overexpressing ductal carcinoma *in situ* (DCIS) prior to surgical resection (123). The DC vaccine was loaded with HER2 MHC class-I and class-II peptides and activated *in vitro* with IFN-γ and bacterial lipopolysaccharides to produce cytokine IL-12. The 13 patients enrolled in the study showed high rates of HER2-specific sensitization for both IFN-γ-secreting CD4^+^ T cells (85%) and CD8^+^ T cells (80%) and induction of tumor-lytic antibodies. Interestingly, 7 patients demonstrated markedly decreased HER2 expression in surgical tumor specimens, suggesting a possible immunoediting process for HER2-expressing tumor cells. A follow-up trial in the neoadjuvant setting involving 54 HER2-positive patients with DCIS or early invasive breast cancer indicated that clinical and immune responses to the tumor did not vary significantly between different routes (intralesional versus intranodal versus intralesional-plus-intranodal) by which the same DC vaccine is administered (110).

In another clinical study, 7 patients with stage II-IV HER2-overexpressing breast cancer were injected with autologous DCs pulsed with a peptide derived from the intracellular domain of HER2 after surgery and adjuvant therapy (124). HER2-specific antibodies were detected in six patients, and all of the seven participants were disease-free at a median follow-up of 5 years.

Clinical trials involving DC-based vaccines are moving forward currently. These trials use DCs pulsed with HER2-derived peptide E75 plus trastuzumab and vinorelbine (NCT00266110), and DCs pulsed with HER2 peptides 369-377 and 689-697 (NCT00923143).

### 4.2 Vaccines Targeting Non-HER2-Related Antigens

Besides HER2 or HER2-related peptides, non-HER2-related antigens are also studied in vaccination for breast cancer, indicating opportunities of using cancer vaccines to treat HER2-negative breast cancers. Mucins, human telomerase reverse transcriptase (hTERT), and p53 are some of the studied targets. Next, breast cancer vaccines targeting non-HER2-related antigens will be introduced in the order of their types.

#### 4.2.1 Carbohydrate Antigen Vaccine—Sialyl-Tn

Theratope^®^, the STn-keyhole limpet hemocyanin (KLH) vaccine, is a synthetic STn conjugated to the KLH carrier protein. A significantly higher antibody level was observed in patients pretreated with a low dose of cyclophosphamide and vaccinated with STn-KLH in a randomized phase 2 trial (111). In the following double-blinded phase 3 study, a total of 1028 metastatic breast cancer patients across 126 centers in 10 countries were randomized to receive the STn-KLH vaccine or only KLH alone. Patients in both arms also received a low dose of cyclophosphamide (300 mg/m^2^) to increase the immunogenicity of the vaccine. The primary endpoint was time to progression (TTP) and overall survival (OS). Despite the fact that significant antibody titers specific for STn were produced in patients treated with the vaccine, no significant improvement in TTP or OS was observed in the trial (101). The TTP was 3.4 months in the treatment group and 3.0 months in the control group (P=0.353). The median survival time was 23.1 months and 22.3 months (P=0.916), respectively, in the treatment and control groups. Lack of more strict eligibility criteria might be part of the reason for the negative results in that only 30%-40% of the breast cancer express STn, and no detection of STn expression was performed on the patients enrolled in the study (125). A subgroup analysis showed that the vaccinated arm had longer TTP and OS compared with the control arm in patients receiving endocrine therapy, indicating using the STn-KLH vaccine in combination with the endocrine therapy might improve clinical outcomes (126).

#### 4.2.2 Peptide Vaccine—hTERT

The hTERT is nearly universally overexpressed in human cancers, including breast cancer, and it can be recognized by CD8^+^ T cells. Nineteen patients with metastatic breast cancer received hTERT peptide vaccination, and high hTERT-specific CD8^+^ T cell responses were induced after vaccination in 9 participants (114). An exploratory analysis revealed that the median OS was significantly longer in the patients who achieved an immune response to hTERT compared with those who did not. Trials evaluating hTERT vaccines are underway in the metastatic setting (NCT00573495 and NCT01660529) and the adjuvant setting (NCT02960594 and NCT00753415).

#### 4.2.3 DNA-Based Vaccine—MUC-1, Mammoglobin-A

PANVAC is a recombinant poxviral-vector cancer vaccine consisting of a priming dose with recombinant vector and subsequent doses with recombinant fowlpox vector. Each vector encodes the transgenes for CEA and MUC-1 and transgenes for 3 human co-stimulatory molecules (B7.1, ICAM-1, and LFA3). In a phase 2 clinical trial, 48 patients with metastatic breast cancer of all subtypes were randomized to receive PANVAC plus docetaxel or docetaxel alone (112). A trend towards improvement in progression-free survival (PFS) was detected. The median PFS in the vaccinated arm was 7.9 months compared with 3.9 months in the control arm (P=0.09) at the median potential follow-up of 42.8 months.

Mammoglobin-A (MAM-A) is another breast cancer-associated antigen overexpressed in 40% to 80% of primary breast cancers (127). A phase I clinical trial of a MAM-A DNA vaccine was initiated to evaluate its safety and efficacy. In this study enrolling 14 patients with stable metastatic breast cancer, significant increase in the frequency of MAM-A specific CD8^+^ T cells and no severe adverse events were observed after vaccination. Exploratory analysis also suggested an improved 6-month PFS rate in the vaccinated patients compared with those who met all eligibility criteria but were not vaccinated because of HLA phenotype (53% vs. 33%, P=0.011) (99).

#### 4.2.4 DC-Based Vaccine—p53

The efficacy of a DC-based vaccine loaded with wild-type p53-derived peptide and stimulated with IL-4 and GM-CSF has been evaluated. This vaccine was administered in combination with low-dose IL-2 to 26 metastatic breast cancer patients in the study (113). Seven patients discontinued vaccination due to rapid disease progression or death. Eight of nineteen evaluable patients attained stable disease or minor regression while the rest of the patients had progressive disease, indicating the effect of p53-specific immune therapy. Surprisingly, the frequency of Treg cells was found to be almost doubled after vaccination in the analysis (128).

#### 4.2.5 DC-Tumor Cell Fusion Vaccine—Multiple Antigens

A phase I clinical trial evaluated the fusion cell vaccination in patients with metastatic breast cancer and renal cancer (115). A total of 32 breast cancer patients were enrolled in the study and vaccine generation was successful in 16 patients. Among them, 6 patients were withdrawn from the study before receiving the vaccine due to significant disease progression. The rest of the patients were vaccinated with autologous fusion cells. As a result, no significant treatment-related toxicity or autoimmunity was observed. Two patients exhibited disease regression and 1 patient had disease stabilization.

## 5 Combinational therapy of breast cancer vaccine

ICB has reformed the treatment strategy for some solid tumors, including melanoma and non-small cell lung cancer. As for breast cancer, ICB has already demonstrated its efficacy in treatment for metastatic TNBC (7). However, the addition of ICB to trastuzumab did not show a clinically significant improvement in PFS for HER2-positive metastatic breast cancer and was associated with more adverse events (60). Currently, an area of active investigation is combining the vaccine with ICB to overcome cancer tolerance. As mentioned previously, ICB makes the effector immune cells able to attack the tumor cells by blocking the inhibitory receptors such as PD-1/PD-L1 and CTLA-4 (59). Results of some preclinical studies indicate that tumor vaccines will also upregulate the expression level of inhibitory receptors on the cell surface when activating T cells (129). One underlying mechanism is that increased IFN-γ secreted by tumor-specific T cells can correspondingly upregulate the expression of PD-L1 on tumor cells and APCs, which is set initially to prevent over-amplification of the immune reactions occurring in the body (130). Therefore, the administration of ICB can probably relieve the immunosuppressive effect that attenuates anti-tumor immunity induced by vaccines. The combined use of breast cancer vaccine and ICB represents a promising strategy that may potentially enhance and prolong the duration of the immune response and ultimately lead to significant clinical benefits.

Additionally, applying cancer vaccines in combination with established therapies might also improve efficacy. Growing evidence has shown that some HER2-derived peptide vaccines may work synergistically when combined with anti-HER2 monoclonal antibodies (131). Studies indicates that chemotherapy and radiation therapy are associated with immunogenic cell death (132). Such immune response might help induce durable immune response when the therapies are applid in combination with cancer vaccines. Consistently, the effect of combining cancer vaccination with chemotherapy, targeted therapy, endocrine therapy, and even radiation therapy are also worth exploring (104, 133, 134).

## 6 Conclusion and Future Perspective

Active vaccination therapy for breast cancer has several theoretical advantages compared to conventional chemotherapy and anti-HER2 immunotherapy *via* monoclonal antibodies: better tolerance, lower toxicity, and long-lasting immune response with tumor specificity (64). In addition, some vaccines can elicit immunity to tumors without any HER2 expression if the vaccine target is derived from non-HER2-related antigens.

However, clinical trials evaluating breast cancer vaccines have provided limited evidence of clinical benefits despite the successful induction of immune responses. It was demonstrated that the prognosis of patients who received vaccination is usually associated with the degree of immune responses (114). And in the initial E75 Phase 2 trial, immunity was noted to wane with time, and this corresponded with increased recurrences noted in the vaccine arm (103). Therefore a potential explanation for negative results to date is that the effective anti-tumor immunity stimulated by vaccines is not long-lasting enough to produce significant benefits in survival. The reason why the anti-tumor immune response fades so early may be attributed to the following factors: suboptimal vaccine formulations, the immune tolerance developed to specific tumor antigens, and the immune-suppressive microenvironment. Early trials have acknowledged that a HER2-specific MHC class-I peptide epitope vaccine alone elicits only short-lived CD8^+^ T cell responses (135). In fact, as previously described, pre-existing immunity against HER2 has been detected in some patients. Nevertheless, the natural immune response is not strong enough to cultivate significant benefits due to immune tolerance. The immune tolerance that gradully builds in a long-term process might be a key factor to both the pre-existing immunity and the decreased immunity stimulated by vaccine. Hence, how to suppress immune tolerance for long and how to effectively exploit the natural immune response in the patients remains vital challenges to improve efficacy of breast cancer vaccines. Additionally, throughout the immunoediting process, the immunosuppressive effect will gradually outweigh the anti-tumor immunity as the tumor progresses. Even though the cancer vaccines manage to enhance the ability of the immune system to recognize specific tumor antigens, the effector immune cells such as CTLs might be incapable of efficiently eradicating the tumor cells in an immunosuppressive TME.

To overcome this issue, the optimal immunization dose and schedule, delivery routes, and choices of immunologic boosters need to be investigated. It was demonstrated that booster inoculations could maintain immunity, and those who received scheduled booster inoculations were less likely to recur (136). Moreover, the results of different peptide vaccines indicate that vaccine formulations should be tailored to the features of the tumor being targeted. Tolerance might be avoided by using subdominant epitopes with lower binding affinity against antigens with higher expression levels. For instance, E75, a dominant epitope of HER2, appears most effective in tumors expressing low degrees of HER2, while GP2, a subdominant epitope of HER2, shows more potential in HER2-overexpressing breast cancer in combination with trastuzumab. AE37, the MHC class-II targeted vaccine, shows the greatest efficacy in TNBC and may be helpful in all HLA subtypes (137).

The immune system maintains the delicate balance in our body to effectively remove non-self antigens and prevent autoimmune diseases at the same time. Despite the various obstacles that we encountered in the development of the breast cancer vaccine, the concept behind cancer vaccines that autologous immune systems can be mobilized to fight cancers has never been abandoned. Although the current results of clinical trials evaluating breast cancer vaccines are not satisfying, we believe novel strategies will eventually lead to improved efficacy as our understanding of cancer immunology deepens.

## Author Contributions

All authors wrote, read and approved the final manuscript.

## Funding

Supported by grants from the National Natural Science Foundation of China (82072916), the 2018 Shanghai Youth Excellent Academic Leader, the Fudan ZHUOSHI Project, Chinese Young Breast Experts Research project (CYBER-2021-A01).

## Conflict of Interest

The authors declare that the research was conducted in the absence of any commercial or financial relationships that could be construed as a potential conflict of interest.

## Publisher’s Note

All claims expressed in this article are solely those of the authors and do not necessarily represent those of their affiliated organizations, or those of the publisher, the editors and the reviewers. Any product that may be evaluated in this article, or claim that may be made by its manufacturer, is not guaranteed or endorsed by the publisher.
